# A comparative correlational study of coping strategies and quality of life in patients with chronic heart failure and the general Swedish population

**DOI:** 10.1002/nop2.81

**Published:** 2017-04-18

**Authors:** Annika Nilsson, Marianne Carlsson, Ragny Lindqvist, Marja‐Leena Kristofferzon

**Affiliations:** ^1^ Department of Health and Caring SciencesUniversity of GävleGävleSweden; ^2^ Section of Caring SciencesDepartment of Public Health and Caring SciencesUppsala UniversityUppsalaSweden

**Keywords:** chronic heart failure, coping, cross sectional survey, general Swedish population, Jalowiec Coping Scale, quality of life

## Abstract

**Aim:**

The aim was to compare coping strategies and quality of life (QoL) in patients with chronic heart failure (CHF) with such strategies and QOL in persons from two general Swedish populations and to investigate relationships between personal characteristics and coping strategies.

**Design:**

A cross‐sectional, comparative and correlational design was used to examine data from three sources.

**Methods:**

The patient group (*n *=* *124), defined using ICD‐10, was selected consecutively from two hospitals in central Sweden. The population group (*n *=* *515) consisted of persons drawn randomly from the Swedish population. Data were collected with questionnaires in 2011; regarding QoL, Swedish population reference data from 1994 were used.

**Results:**

Overall, women used more coping strategies than men did. Compared with the general population data from SF‐36, patients with CHF rated lower QoL. In the regression models, perceived low “efficiency in managing psychological aspects of daily life” increased use of coping. Other personal characteristics related to increased use of coping strategies were higher education, lower age and unsatisfactory economic situation.

## 1 Introduction

Chronic heart failure (CHF) is a worldwide problem and population‐based studies in North America, Europe and Asia have shown that 1%–2% of the population live with the disease (Ponikowski et al., 2014). A report from the American Heart Association estimates that the prevalence of CHF will increase by 46% from 2012–2030 (Mozaffarian et al., 2015). A case control study in 52 countries showed that nine modifiable risk factors (e.g. smoking, hypertension, diabetes, obesity, physical inactivity, unhealthy diet, psychosocial stress) account for most heart attacks, independent of age, gender and ethnic group (Yusuf et al., 2004). These risk factors are the same for all cardiovascular diseases, including CHF (McMurray et al., 2012). Factors contributing to the increase in CHF are ageing populations, shifting toward a Western‐type lifestyle and the fact that, today, more patients with heart attacks and other cardiovascular diseases are surviving. In regions with previously low levels of CHF such as parts of Latin America and Japan the numbers of persons with CHF are also increasing (Ponikowski et al., 2014).

Living with chronic heart failure (CHF) involves different symptoms such as breathlessness on exertion or during rest, peripheral oedema and fatigue. Some patients with CHF lose confidence in their physical abilities and in their ability to function in their social network (Jeon, Kraus, Jowsey, & Glasgow, 2010). Therefore, they need relevant coping strategies to help them deal with daily life (Aldwin, 1994). According to Lazarus and Folkman (1984), coping is a complex process, where individuals' perception of stress reflects the relationship between their experiences in their environment and their available resources. In their view, perceived stressful events, such as threats, challenges or various forms of harm, require emotion‐focused coping to regulate emotions or problem‐focused coping to deal with the problem causing the distress.

The coping process results in various event outcomes—favourable resolution, unfavourable resolution or no resolution—and emotions are generated in connection with appraising events, coping with them and in relation to event outcomes (Lazarus & Folkman, 1984). The efficiency of different coping strategies varies across situations, but a problem‐focused strategy is often more effective than an emotion‐focused one (Lazarus, 1993). This has also been reported in a study among patients with CHF, where problem‐focused coping strategies predicted positive emotions (Nahlén & Saboonchi, 2010). Patients with CHF have a complex situation, in that they must deal with different symptoms as well as physical and social limitations. This poses threats to health, well‐being and quality of life (QoL) (Hwang, Liao, & Huang, 2014; Nieminen et al., 2015).

### 1.1 Background

CHF is one of the diseases that has great effects on QoL (Jaarsma, 2005), which include the physical, psychological and social dimensions as well as well‐being (Kinney, Burfitt, Stullenbarger, Rees, & Read, 1996). Persons with CHF often struggled with problems such as fatigue, sleep disturbances, helplessness, frustration, uncertainty and restrictions related to physical functioning and work. These problems have a major impact on their daily life in the form of social isolation, living in fear and losing their sense of control (Jeon et al., 2010). Strömberg and Mårtensson (2003) found that physical and social restrictions in daily life were more troublesome for men. For women, restrictions in their ability to support family and friends were most difficult to accept.

Many patients with CHF have difficulties adhering to their medication regimen, diet and fluid restrictions and incorrectly interpret the symptoms and signs of CHF. Co‐morbidity disorders are common in these patients, which increases the difficulty of interpreting symptoms. Patients with CHF may also suffer from cognitive dysfunctions and depression, which may affect their ability to take in knowledge about the disease and recommended lifestyle changes (Jaarsma, 2005; Spaling, Curie, Strachian, Harkness, & Clark, 2015). Several studies (Mendes de Leon et al., 2010; Park et al. 2012, Perez‐ Garcia, Oliván, & Bover, 2013) have emphasized that a large proportion of persons with CHF live with depressive symptoms, which influence their QoL negatively. Patients with CHF (Heo, Moser, & Widener, 2007; Juenger et al., 2002; Lesman‐Leegte et al., 2009) have rated significantly lower QoL compared with persons from various populations (Juenger et al., 2002; Lesman‐Leegte et al., 2009). Some studies have found that, compared with men, women with CHF rate themselves as having lower QoL (Friedman, 2003; Perez‐ Garcia et al., 2013), even after adjusting for age and number of diseases (Lesman‐Leegte et al., 2009). In contrast, Heo et al., (2007) reported no differences in QoL between women and men with CHF.

Problem‐focused coping has been shown to predict higher psychological QoL among patients with CHF (Hopp, Thornton, Martin, & Zalenski, 2012; Perez‐ Garcia et al., 2013), whereas avoidant coping strategies have been shown to be related to poorer physical functioning (Eisenberg, Shen, Schwarz, & Mallon, 2012). In both general populations (Folkman & Lazarus, 1980; Lindqvist, Carlsson, & Sjödén, 2000; Matud, 2004) and among patients with chronic heart and kidney diseases (Kristofferzon, Lindqvist, & Nilsson, 2011), women tended to use more coping strategies than men did and older persons tended to use fewer coping strategies than younger persons did (Folkman & Lazarus, 1980; Lindqvist et al., 2000; Matud, 2004). A significant correlation was also found between higher education and more use of both problem‐ and emotion‐focused coping among adults with chronic kidney diseases (Harwood, Wilson, & Sontrop, 2011).

To summarize the literature, studies using qualitative and quantitative approaches have shown that persons with CHF struggle with various problems that have an impact on their daily life and that influence their QoL. Previous studies have revealed that problem‐focused coping is associated with greater well‐being and that women generally use more coping strategies than men do. Some studies have reported relationships between personal characteristics and coping in patients with CHF. We have not found any previous study comparing patients with CHF and a general population with regard to coping. Concerning QoL, two studies (Juenger et al., 2002; Lesman‐Leegte et al., 2009) have compared patients with CHF with gender‐matched community controls.

Owing to the complexity of the disease and its consequences, health care providers need to work in teams with long‐term management to support patients' self‐care, including follow‐up and monitoring. The CHF nurse has an important role to play in helping the patient and family cope with symptoms, treatment, medication side effects and lifestyle changes (Jaarsma, 2005; Ponikowski et al., 2014). Thus, the CHF nurse needs to have adequate information about the patient's personal characteristics and what coping strategies the patient uses to manage the consequences of illness. Effective coping strategies can increase the patient's ability to provide self‐care, which can lead to more independence, less hospital care and have a positive impact on the patient's QoL. This research contributes to the exciting body of knowledge surrounding coping and QoL since few studies have compared coping strategies and QoL in patients with CHF and in general populations. Furthermore, understanding coping mechanisms in patients with CHF and how they vary from the general population, may provide insights that could further enhance the delivery of responsive healthcare services that in turn optimizes patient and health system outcomes. The present study compared coping strategies and QoL in patients with CHF and in persons from two general Swedish populations. A further aim was to investigate possible relationships between personal characteristics and coping strategies.

## 2 Methods

### 2.1 Setting, samples and data collection

The study is a cross‐sectional, comparative and correlational in design. Data from three sources were used: questionnaires from patients with CHF and from a Swedish population group and reference data from a general Swedish population.

Inclusion in the patient group was based on the International Statistical Classification of Diseases and Related Health Problems, 10th Revision, in 2011 and patients were selected from two hospitals in central Sweden. Patients meeting the following inclusion criteria were asked to participate: 18–85 years of age, CHF >3 months and understands the Swedish language. One hundred and twenty‐four out of 287 patients participated (40%); 140 did not answer the questionnaire and 23 declined to participate. Of the 124 patients, 113 (91%) were born in Sweden.

The inclusion criteria for the population group in 2011 were: 18–85 years of age and understands the Swedish language. This group consisted of persons drawn from the entire Swedish population, including all parts of the country. The random selection was based on the inclusion criteria and was carried out by Infodata AB. From the postal codes, Infodata AB divided the population into 5‐year age intervals. Of the 2500 persons, 798 completed the questionnaire (32%) and of the 515 individuals who participated in the analyses, 453 (88%) had been born in Sweden.

Regarding QoL, SF‐36 (1994) data from a general Swedish population were used for comparison with the patient group. The data were from 1994 because we could not find any more recent data. Two questionnaires (coping and QoL) were administered to patients with CHF in 2011. The coping questionnaire was sent to a random sample from the general Swedish population in 2011.

### 2.2 Measurements

Two standardized questionnaires were used: Jalowiec Coping Scale 60 (JCS‐60) (Jalowiec, 1988, 1994; Jalowiec, Murphy, & Powers, 1984) and The Short‐Form 36 Health Survey (SF‐36) (Persson, Karlsson, Bengtsson, Steen, & Sullivan, 1998; Sullivan, Karlsson, & Ware, 1994; Ware & Gandek, 1998). In addition, data were collected on personal characteristics such as age, civil status, educational level, economic situation, working conditions, living area and perceived efficiency in managing the physical, psychological, social and existential aspects of daily life, such as life values. The personal characteristics and the response alternatives are presented in Table 1.

**Table 1:** Personal characteristics of the patient and general Swedish population group (2011)

Characteristics	Patient group (*n *=* *124)		Population group (*n *=* *515)	
	Women (*n *=* *28)	Men (*n *=* *96)	Women (*n *=* *269)	Men (*n *=* *245)
Age	*M/SD*	*M/SD*	*M/SD*	*M/SD*
	71/9.92 (49–84)	70/8.25(46–85)	63/10.48 (46–85)	64/10.14 (46–85)
Civil status	Number/%	Number/%	Number/%	Number/%
Married/cohabiting	20/71	62/65	174/65	205/84
Single	7/25	32/33	**91** 1/34	37/15
Educational background				
Compulsory school	11/39	58/60	74/27	81/33
Senior high school	9/32	25/26	90/34	88/36
University	7/25	10/10	103/39	75/31
Economic situation				
Very good	3/11	8/8	39/15	29/12
Good	8/29	31/32	117/44	126/51
Acceptable	14/50	46/48	92/34	73/30
Unsatisfactory	2/7	8/8	13/5	12/5
Very unsatisfactory	0/0	1/1	6/2	5/2
Work condition				
Working	2/7	15/16	118/44	118/49
Retired	23/82	78/81	123/46	109/45
Other	2/7	1/1	27/10	15/6
Living area				
Urban	10/36	26/27	61/23	63/26
Middle‐sized town	7/25	24/25	52/19	55/22
Small town	5/18	13/14	66/25	41/17
Rural	5/18	32/33	89/33	85/35
How often do you exercise/week (>30 min)				
0 days	12/43	30/31	27/10	43/18
1–3 days	8/29	34/35	123/46	120/49
4–5 days	3/11	18/19	65/24	45/18
6–7 days	4/14	11/12	53/20	36/15
Comorbidities				
Heart failure	28/100	96/100	8/3	22/9
Renal failure	4/14	8/8	3/1	3/1
Diabetes mellitus	4/14	21/22	11/4	26/11
Chronic obstructive pulmonary disease	4/14	7/7	7/3	8/3
Rheumatoid arthritis	7/25	7/7	**23/9**	6/2
Parkinson's	0/0	0/0		1/4
Chronic pain	4/14	15/16	39/15	19/9
MS	0/0	0/0		3/1
Obesity	3/11	11/12	15/6	11/5
Cancer	3/11	5/5	16/6	18/7
Gastrointestinal symptoms	6/21	12/13	38/14	24/10
	*M/SD*	*M/SD*	*M*	*M/SD*
Perceived efficiency in managing (1 = very bad to 5 = very good)				
Physical aspects (e.g. pain, tiredness)	2.71/.85	2.88/.96	3.84/.85	3.78/.83
Psychological aspects (e.g. anxiety, anger)	3.04/.94	3.47/.85	3.70/.86	3.67/.88
Social aspects (e.g. family, friends)	3.82/.91	3.85/.73	4.14/.76	4.03/.73
Existential aspects (life values)	3.43/.92	3.63/.72	**4.04** 4 **/**.77	3.84/.85

Bold figures; Chi‐square tests; ^1^p = .001, ^2^p = .009, ^3^p = .003; Mann‐Whitney U test ^4^p = .007.

The JCS‐60 (Jalowiec, 1988) consists of eight coping scales designed to measure problem‐ and emotion‐focused coping. The coping scales are called: (i) Confrontative (confronting the problem, 10 items); (ii) Evasive (avoiding the problem, 13 items); (iii) Optimistic (positive thinking about the problem, 9 items); (iv) Fatalistic (feeling that it is useless to try to do anything at all, 4 items); (v) Emotive (responding in an emotional way, 5 items); (vi) Palliative (doing things to feel better, 7 items); and (vii) Supportive (using different sources of support, 5 items) and (viii) Self‐reliant (depending on oneself, 7 items). A 4‐point rating scale was used to assess how often each coping strategy was used (0 =  never, 1 =  rarely, 2 =  sometimes, 3 = often) (Jalowiec, 1988, 1994; Jalowiec et al., 1984). Mean values were calculated for each subscale as well for the total scale. In the present study, the internal consistency of the JCS‐60 scales varied from α = 0.46 (Emotive) to 0.88 (Confrontative) for the patients and from α = 0.54 (Supportive) to 0.88 (Evasive) for the persons in the general Swedish population (2011).

The SF‐36 (Sullivan et al., 1994) was used to measure QoL. It consists of eight scales including: (i) Physical functioning (PF, 10 items); (ii) Role‐physical (RP, 4 items measuring role limitations due to physical problems); (iii) Bodily pain (BP, 2 items); (iv) General health (GH, 5 items), (v) Vitality (VT, 4 items); (vi) Social functioning (SF, 2 items); (vii) Role‐emotional (RE, 3 items measuring role limitations due to emotional problems); and (viii) Mental health (MH, 5 items). The response alternatives used were yes/no, 3‐grade (1 = yes, greatly limited, 3 = no, not at all limited), 5‐grade (e.g. 1 = not at all, 5 = very much) and 6‐grade scales (1 = all the time, 6 = never of the time). The scores for each scale were coded, summed and transformed into a scale ranging from 0 (worst possible health) to 100 (best possible health). The SF‐36 has been shown to have good reliability and validity (Persson et al., 1998; Ware & Gandek, 1998). The internal consistency of the SF‐36 scales in the present study varied between α = 0.75 and 0.91 for the patient group.

### 2.3 Data analysis

All analyses were performed using SPSS, version 20. When between‐ and within‐group differences were tested in coping and QoL, the chi‐square test was used for categorical variables and the Mann‐Whitney U‐test for continuous variables. In the analyses, personal characteristics were used as independent variables. A non‐parametric test for continuous variables was used owing to the small sample size in the patient group (Ware & Gandek, 1998). To obtain comparable reference data for coping, the patients were matched for age and sex with persons from the Swedish population in 2011. To obtain comparable reference data for QoL, the patients were matched for age and sex with general Swedish population data from 1994. The variable age was divided into 5‐year age groups (46–50, 51–55, 56–60, etc.). In the comparisons, patients were compared with the mean value for their age group. For the JCS‐60, mean value substitution based on data from the patient group and from a general Swedish population was used when respondents had answered at least 50% of the items. Missing values for SF‐36 were substituted, as recommended in the manual (Sullivan et al., 1994). Cronbach's alpha was used to measure the instruments' internal consistency.

The JCS ‐60 (Jalowiec, 1988, 1994; Jalowiec et al., 1984) is based on the theoretical concepts of Lazarus and Folkman (Lazarus & Folkman, 1984) and consists of eight coping scales designed to measure two coping strategies: problem‐focused and emotion‐focused coping. The eight scales in the JCS‐60 were recoded to create two composite variables. The scales optimistic, self‐reliant, confrontative and supportive were recorded to create “problem‐focused coping” (α = .94) and the scales evasive, fatalistic, emotive and palliative were recoded to create “emotion‐focused coping” (α = .88). To test bivariate correlations between personal characteristics and problem‐ and emotion‐focused coping, Pearson′s and biserial correlations were performed. Correlations between the variables were appraised to be weak (r = 0–.2), moderate (r = .3–.6) or strong (r = .7–1) (Brace, Snelgar, & Kemp, 2012). To test for regression model assumptions, linear regression analyses (method enter) were used between personal characteristics (independent variables) and problem‐ and emotion‐focused coping strategies (dependent variables). All variance inflation factor (VIF) values were below 2 (Tabatchnick & Fidell, 2007). The statistical significance level was set to p < .05 (two‐tailed).

### 2.4 Ethical considerations

The study conformed to the ethical principles defined in the World Medical Association Declaration of Helsinki (WHO 2001) and it was approved by the regional ethical review board (reg. no. 2010/346). The questionnaires, a cover letter with information about the study and a stamped reply envelope were sent by mail to the persons in the two samples. Two reminders were sent out and all data were coded using a subject number. The questionnaires were kept separate from the code list and locked in a special archive for research data at the university. When respondents completed and returned the questionnaires, this was considered to constitute their informed consent. Only two of the authors (AN, M‐LK) knew the identity of the persons. All study participants were guaranteed confidentiality and were informed that their participation was voluntary (The Swedish Research Council 2011).

## 3 Results

### 3.1 Characteristics of the patient group and the Swedish population group (2011)

One hundred and twenty‐four patients participated: 28 women and 96 men between 46–85 years of age (mean age = 73, *SD* 9.41). No significant differences with regard to age and gender were found between patients who participated (mean age = 70, *SD* 8.65; 24% women) and those who did not participate (mean age = 71, *SD* 11.52; 23% women). In the population group, 798 out of 2500 persons completed the questionnaires (32%): 448 women and 349 men between 18–85 years of age (mean age = 53, *SD* 17.26).

The characteristics of the patient group and the population group are presented in Table 1. In the patient group, no significant differences between women and men were found in relation to the personal characteristics, while in the population group several differences were found. Women were more likely to be single (p = .001), exercised more often (p = .009), were more likely to report rheumatoid arthritis (p = .003) and perceived more self‐efficiency in managing existential aspects of daily life (p = .007).

### 3.2 Between‐ and within‐group differences in coping strategies and QoL

Between‐group differences in coping showed that the patient group (*n *=* *121) used more evasive (p < .000), optimistic (p < .000) and fatalistic coping (p < .025) than the population group did (*n *=* *121), whereas persons in the population group (2011) used more confrontative (p < .000) and emotive coping (p < .000) (not in Table 2).

**Table 2:** Between‐ and within‐group differences in coping among women (*n *=* *27) and men (*n *=* *94) with CHF and a general Swedish population group (2011) (*M* = mean; *SD* = standard deviation)

Instrument and scales	Patient group	Population group	p‐values
JCS‐60^a^	*M/SD*	*M/SD*	Woman
Confrontative	1.69/.68	1.60/.36	.526
Optimistic	2.08/.48	1.88/.17	**.017**
Self‐reliant	1.69/.57	1.57/.20	.096
Fatalistic	1.35/.48	1.16/.14	**.004**
Evasive	1.34/.43	1.01/.13	**.000**
Emotive	.81/.46	.80/.17	.481
Supportive	1.37/.64	1.12/.22	.097
Palliative	.99/.47	.92/.16	.208
Problem‐focused	1.71/.45	1.54/.23	**.027**
Emotion‐focused	1.12/.33	.97/.14	**.022**
Men			
Confrontative	1.23/.72	1.61/.24	**.000**
Optimistic	1.80/.68	1.68/.18	**.000**
Self‐reliant	1.46/.72	1.55/.21	.942
Fatalistic	1.13/.68	1.00/.07	.371
Evasive	1.07/.58	.93/.21	**.020**
Emotive	.59/.57	.70/.01	**.000**
Supportive	.88/.63	.87/.15	.919
Palliative	.83/.54	.73/.15	.422
Problem‐focused	1.35/.60	1.43/.15	.788
Emotion‐focused	.91/.49	.84/.15	.363
Within‐group differences; p‐ values			
Confrontative	**.007**	.133	
Optimistic	.081	**.000**	
Self‐reliant	.183	.407	
Fatalistic	.125	**.000**	
Evasive	**.047**	.096	
Emotive	**.011**	**.009**	
Supportive	**.001**	**.000**	
Palliative	.110	**.000**	
Problem‐focused	**.003**	**.015**	
Emotion‐focused	**.000**	**.000**	

Mann‐Whitney U‐test was used for the comparisons.

Bold figures = p < .05.

a JCS‐60: 0 = never used, 3 often used.

Table 2 shows that women in the patient group used more optimistic, fatalistic, evasive as well as problem‐ and emotion‐focused coping than did women in the population group. Men in the patient group used more optimistic and evasive coping than men in the population group did, whereas men in the population group used more confrontative and emotive coping. Within‐group differences showed that women in both groups used more problem‐ and emotion‐focused coping than men did (Table 2).

Persons in the patient group (*n *=* *109–122) rated lower QoL on all scales (p < .000), except for the Role emotional scale, compared with the general Swedish population data (1994) (*n *=* *109–122) (not in Table 3). Table 3 shows that, for women in the patient group, QoL was lower on six of the scales compared with women in the population data. For men in the patient group, QoL was lower on seven of the scales compared with men in the population data. Within‐group differences showed that women in both groups rated lower QoL than men did (Table 3).

**Table 3:** Between‐ and within‐group differences in QoL among women (*n *=* *28) and men (*n *=* *94) with CHF and a general Swedish population group (1994) (*M* = mean; *SD* = standard deviation)

Instrument and scales	Patient group	Reference data	p‐values
SF‐36^a^	*M/SD*	*M/SD*	Woman
Physical function (PF)	39.1/20.9	63.8/11.0	**.000**
Role physical (RP)	24.3/35.8	56.6/14.1	**.000**
Bodily Pain (BP)	52.2/28.4	63.7/4.1	.098
General health (GH)	34.0/18.7	60.8/7.3	**.000**
Vitality (VT)	38.9/22.6	57.0/9.6	**.001**
Social function (SF)	60.7/31.5	80.3/5.1	**.010**
Role emotional (RE)	55.6/41.3	69.0/10.4	.616
Mental health (MH)	63.0/20.3	74.6/4.4	**.015**
Men			
Physical function (PF)	50.8/27.6	70.4/8.2	**.000**
Role physical (RP)	40.1/43.1	61.3/9.7	**.001**
Bodily Pain (BP)	59.0/28.4	68.8/2.7	**.001**
General health (GH)	45.3/21.2	66.7/2.3	**.000**
Vitality (VT)	46.4/24.7	64.7/4.2	**.000**
Social function (SF)	72.6/25.8	85.5/3.2	**.002**
Role emotional (RE)	58.4/43.3	74.6/7.3	.346
Mental health (MH)	72.6/18.2	82.7/.6	**.001**
Within‐group differences; p‐ values			
Physical function (PF)	.053	**.000**	
Role physical (RP)	.117	**.024**	
Bodily Pain (BP)	.227	**.000**	
General health (GH)	**.036**	**.000**	
Vitality (VT)	.303	**.007**	
Social function (SF)	.084	**.000**	
Role emotional (RE)	.730	**.025**	
Mental health (MH)	**.019**	**.000**	

Mann‐Whitney U‐test was used for the comparisons.

Bold figures = p < .05.

a SF‐36: 0 = lowest perceived QoL, 100 = highest perceived QoL.

### 3.3 Relationships between personal characteristics and coping strategies in the patient group and in the Swedish population group (2011)

Table 4 shows a Pearson's correlation test between group characteristics (independent variables) and problem‐ and emotion‐focused coping (dependent variables). The range of significant correlations varied from r = .14 to r = −.50. The highest correlation was found between perceived efficiency in managing psychological aspects of daily life and emotion‐focused coping for men in the patient group as well as for women and men in the population group.

**Table 4:** Bivariate correlations between problem‐ and emotion‐focused coping as dependent variables and personal characteristics as independent variables

Groups and DVs	Age	Education	Economic situation	Physical	Psycho‐logical	Social	Existential
Patient group							
Women (*n *=* *27)							
Problem‐focused	−.408*		.399*				
Emotion‐focused							
Men (*n *=* *94)							
Problem‐focused			−.209*	−.209*	−.319**	−.214*	−.233*
Emotion‐focused			−.295**	−.303**	−.501**	−.344**	−.380**
Population group							
Women (*n *=* *269)							
Problem‐focused	−.344**	.326**					.141*
Emotion‐focused	−.223**		−.218**	−.158*	−.371**	−.252**	−.154*
Men (*n *=* *245)							
Problem‐focused	−.255**	.275**			−.154*		
Emotion‐focused	−.231**	.156*	−.273**	−.300**	−.423**	−.383**	−.287**

^*^p < .05, ^**^p < .01: DVs = Dependent variables; IVs = Independent variables: Physical aspects (e.g. pain, tiredness), Psychological aspects (e.g. anxiety, anger), Social aspects (e.g. family, friends), Existential aspects (life values), 1 = very bad, 5 very good, Problem‐ and emotion‐focused coping: 0 = never used, 3 = often used.

Table 5 presents the results of the regression analysis for men in the patient group. Significant models emerged that explained 10% of the variance in problem‐focused coping.

**Table 5:** Standard linear regressions with problem‐ and emotion‐focused coping as dependent variables and personal characteristics as independent variables for men in the patient group (*n *=* *89)

Study variables	Standardized coefficient beta	Standard error	95% Confidence intervals	p‐value
Problem‐focused coping	Overall model: adjusted *R* ^*2*^ * *= .097. *F* (2, 35) = 5.13, p‐value < .031			
Age	−.065	.007	−.010 to .018	.556
Education	.223	.091	.007 to .371	**.042**
Economic situation	−.165	.078	−.272 to .036	.133
Physical aspects^a^ (e.g. pain, tiredness)	−.075	.072	−.190 to .097	.522
Psychological aspects^a^ (e.g. anxiety, anger)	−.285	.090	−.372 to −.015	**.034**
Social aspects^a^ (e.g. family, friends)	.014	.112	−.211 to .235	.917
Existential aspects^a^ (life value)	−.028	.113	−.247 to .202	.842
Emotion‐focused coping	Overall model: adjusted R^2^ = .263. F (5, 48) = 6.69, p‐value < .000			
Age	.018	.005	−.010 to .012	.860
Education	.086	.068	−.075 to .196	.379
Economic situation	−.203	.058	−.235 to −.005	**.041**
Physical aspects^a^ (e.g. pain, tiredness)	−.060	.054	−.138 to .077	.572
Psychological aspects^a^ (e.g. anxiety, anger)	−.431	.067	−.376 to −.109	**.001**
Social aspects^a^ (e.g. family, friends)	.000	.084	−.167 to .067	.997
Existential aspects^a^ (life values)	−.078	.084	−.219 to .116	.542

Bold figures = p < .05: ^a^Perceived efficiency in managing physical, psychological, social and existential aspects; the scale ranged from 1 =  very bad to 5 very good, Problem‐ and emotion‐focused coping; 0 =  never used, 3 often used.

Men with a higher education and men who perceived low efficiency in managing psychological aspects of daily life used more problem‐focused coping. The model for emotion‐focused coping explained 26% of the variance, indicating that men who perceived an unsatisfactory economic situation and low efficiency in managing the psychological aspects of daily life used more emotion‐focused coping. No significant model emerged for women in the patient group.

Table 6 displays results of the regression analysis for women and men in the population group. The model for problem‐focused coping explained 17% of the variance for women and 13% of the variance for men. Younger women and men as well as those with a higher education and low efficiency in managing the psychological aspects of daily life used more problem‐focused coping.

**Table 6:** Standard linear regressions with problem‐ and emotion‐focused coping as dependent variables and the personal characteristics as independent variables for women (*W*:* n *=* *260) and men (*M*:* n *=* *243) in the general Swedish population (2011)

Study variables	Standardized coefficient beta	Standard Error	95% Confidence intervals	p‐value
	*W/M*	*W/M*	*W/M*	*W/M*
Problem‐focused coping				
Age	−.228/−.183	.003/.003	−.017 to −.005/−.016 to −.003	**.000/.005**
Education	.256/.211	.038/.041	.086 to .237/.057 to −.218	**.000/.001**
Economic situation	−.061/−.067	.034/.040	−.102 to .032/−.122 to .038	.306/.299
Physical aspects^a^ (e.g. pain, tiredness)	.043/.071	.040/.049	−.054 to .105/−.051 to .141	.532/.352
Psychological aspects^a^ (e.g. anxiety, anger)	−.212/−.216	.044/.046	−.211 to −.038/−0.219 to −.036	**.005/.006**
Social aspects^a^ (e.g. family, friends)	.079/0.011	.047/.058	−.040 to .146/−.107 to .123	.265/.888
Existential aspects^a^ (life values)	.127/0.134	.047/.048	−.009 to .175/−.002 to .189	.078/.055
Overall model for the women	Adjusted *R* ^*2*^ * *= .172, *F* (8, 71) = 12.84, p‐value < .000			
Overall model for the men	Adjusted *R* ^*2*^ * *= .129, *F* (6, 13) = 10.14, p‐value < .000			
Emotion‐focused coping				
Age	−.155/−.179	.003/.002	−.012 to −.002/−.012 to −.003	**.010**/**.003**
Education	.074/.116	.034/.031	−.025 to .109/.001 to .123	.221/**.046**
Economic situation	−.168/−.171	.031/.030	−.147 to −.027/−.148 to −.028	**.005/.004**
Physical aspects^a^ (e.g. pain, tiredness)	−.010/−.113	.036/.037	−.077 to .066/−.131 to .014	.878/.111
Psychological aspects^a^ (e.g. anxiety, anger)	−.322/−.233	.039/.035	−.248 to −.093/−.182 to −.044	**.000/.001**
Social aspects^a^ (e.g. family, friends)	−.044/−.131	.042/.044	−.109 to .057/−.163 to .010	.535/.082
Existential aspects^a^ (life values)	.018/.011	.042/.036	−.072 to .093/−.066 to .077	.797/.882
Overall model for the women	Adjusted *R* ^*2*^ * *= .183, *F* (9, 32) = 11.0, p‐value < .000			
Overall model for the men	Adjusted *R* ^*2*^ * *= .262, *F* (13, 29) = 12.47, p‐value < .000			

Bold figures = p < .05, p < .01, 0 < .001: ^a^Perceived efficiency in managing physical, psychological, social, existential aspects; the Scale range from 1 =  very bad, 5 =  very good. Problem‐ and emotion‐focused coping; 0 =  never used, 3 often used.

The model of emotion‐focused coping explained 18% of the variance for women and 26% for men. Younger women and women who perceived an unsatisfactory economic situation and low efficiency in managing the psychological aspects of daily life used more emotion‐focused coping. Younger men with a higher education and men who perceived an unsatisfactory economic situation and low efficiency in managing the psychological aspects of daily life used also more emotion‐focused coping.

## 4 Discussion

The main results from the present study showed a pattern in relation to coping: women used more problem‐focused as well as emotion‐focused coping strategies than men did. In addition, women in the patient group used coping the most. Compared with the Swedish general population (1994), patients with CHF rated lower QoL. Compared with their male counterparts, women in the patient group rated lower QoL on the dimension's general health and mental health. In the Swedish population data, women showed lower QoL in all domains compared with their male counterparts. In the regression models, perceived low “efficiency in managing the psychological aspects of daily life”, higher education, lower age and an unsatisfactory economic situation were related to increased use of both problem‐ and emotion‐focused coping. No significant models emerged for women in the patient group.

Even if women in the patient group were few in number, the results on coping are in accordance with previous findings (Kristofferzon et al., 2011) as they are for women in the Swedish population group (Lindqvist et al., 2000; Matud, 2004). In the present study, patients with CHF used more problem‐focused than emotion‐focused coping strategies. The most used strategies were optimistic coping (problem‐focused coping) such as hoping that things would get better or trying to think positively. Hopp et al. (2012) showed that common coping strategies used by persons with severe CHF were sharing their illness experiences with others, being flexible in the face of changing circumstances and accepting their physical limitations, all of which can be seen as problem‐focused coping strategies. In previous research among persons with long‐term illnesses, problem‐focused coping has been shown to be associated with better functioning than emotion‐focused coping, which involves strategies such as trying to escape the problem, getting angry or letting off steam (Kershaw, Northouse, Kritpracha, Schafenacker, & Mood, 2004; Taylor & Stanton, 2007; Bucks et al. 2011).

Regarding QoL, patients with CHF, compared with the general Swedish population (1994), rated lower QoL on nearly all of the SF‐36 domains. These findings are consistent with previous investigations (Heo et al., 2007; Juenger et al., 2002; Lesman‐Leegte et al., 2009) indicating that, as a chronic illness, CHF has an impact on QoL. The person must cope with the consequences of the illness and its associated symptoms, treatments and lifestyle changes. In a study by Juenger et al. (2002), persons with chronic diseases (including patients with CHF), compared with a healthy reference group, rated lower QoL on all of the SF‐36 domains. They were also more likely to self‐rate on the lowest end of the physical scale. Regarding the physical scale, in our study, patients with CHF rated just under the middle and lowest QoL on the dimension “role physical”, something also seen in the Swedish population data. However, compared with the men in the patient group, women with CHF rated lower general and mental health. In line with our findings, Juenger et al.'s (2002) study of older persons with CHF showed that women rated lower mental health than men did. For both women and men with CHF, QoL was also reduced compared with a gender‐matched group (Juenger et al., 2002), just as in the present study.

In accordance with the gender differences observed in the present study, several studies including patients with CHF have found that women rated lower QoL and well‐being than men did. A meta‐analytic review (Rutledge, Reis, Linke, Greenberg, & Mills, 2006) showed that female patients reported more emotional distress than male patients did. A systematic review of gender differences (Strömberg & Mårtensson, 2003) showed that life situations for men and women were different, in that women seemed to experience lower QoL than men did, a finding also confirmed by Perez‐ Garcia et al. (2013)

In the regression analyses, one interesting result emerged in all models. Those who perceived low “efficiency in managing the psychological aspects of daily life” used more of both problem‐ and emotion‐focused coping, even though the percentage of explained variance was rather low. Regarding the men with CHF, the result is partly in line with findings from Lindqvist et al.'s studies (Lindqvist, Carlsson, & Sjodén, 1998; Lindqvist, Sjöden, & Carlsson, 2004) of persons with kidney disease. They studied different aspects of handling the illness, showing that the psychological aspect was negatively correlated with emotion‐focused coping. Their explanation was that use of emotion‐focused coping resulted in less successful adaptation to the chronic illness. Yet in our study, the results for men with CHF showed correlations for both emotion‐ and problem‐focused coping. This result can be related to Lazarus and Folkman's revised theoretical model of the coping process, which includes positive psychological states in the process to help persons deal with chronic conditions (Folkman, 1997). The revised model incorporates meaning‐based coping, including both problem‐ and emotion‐focused coping processes. Meaning‐based strategies serve to support use of positive reappraisal (maintain values and beliefs), revise goals, activate spiritual beliefs and positive events in ordinary life, such as having a good dinner, seeing a beautiful flower or being appreciated by a loved one.

In the present study, several patients with CHF had more than one disease, which is in line with previous research, where comorbidities have been shown to be common among persons with CHF (Bennet et al., 2002; Jaarsma, 2005; Ponikowski et al., 2014). In the population group, several of the persons were also dealing with at least one chronic disease (Table 1). It is, however, difficult to compare our results with previous studies because we have found no previous research that compares coping between patients with CHF and the general population group.

Still, it is important that patients comprehend the situation, cope in a constructive way and adhere to essential treatment regimens and recommended lifestyle changes. Research has shown that many patients are unable to integrate their knowledge into daily life and interpret their symptoms as signs of cognitive impairment (Hawkins et al., 2016; Jaarsma, 2005; Spaling et al., 2015), depression, older age and co‐morbidity, which can have a negative impact on QoL (Jaarsma, 2005). The present findings revealed that the patients needed to use more of both problem‐ and emotion‐focused coping to deal with anxiety, fear and anger related to CHF. The clinical importance of these findings is that the CHF nurses need to assess the coping strategies the patients use and how effective they are for their ability to handle the illness, that is, abilities that can be used to improve their self‐management. One approach is to provide person‐centred care, meaning that the nurse takes in account the patient's needs, personal characteristics, preferences, coping strategies and how effective the patient considers these strategies to be. In addition, the care should be related to the patient's context and include significant others. Patients need to be encouraged to test their physical limits to support their sense of control, self‐efficacy and to improve their self‐care skills (Jaarsma, 2005; Spaling et al., 2015).

In future research, use of regression models with larger samples should be considered to explore the relationships between personal characteristics, coping and QoL among persons with chronic illness and to gain deeper insights into how the associations are linked. It is also important to test potential mediating and moderating factors to determine whether they have an impact on coping and QoL. Taylor and Stanton (2007) stated that emotion‐focused coping strategies may be more useful directly after receiving the diagnosis, while problem‐focused strategies may be more useful in the long term. It may be beneficial to investigate meaning‐based coping to see whether it can help explain our results.

### 4.1 Limitations

Some methodological considerations must be pointed out. We used a cross‐sectional design, which did not allow us to look for possible causal relationships. We had a low number of women in the patient group, which makes it difficult to generalize the results to this population of women. There was also a relatively small number of respondents in the population group in 2011. It is difficult to determine why the response rate was low. Possible explanations are that some of the patients who failed to complete the questionnaires were too ill and that people today are tired of participating in surveys. As mentioned before, we sent out two reminders and could not perform an analysis of the non‐responders' personal characteristics, as sample selection was carried out by Infodata AB. Only names and addresses were received for a random sample of the Swedish population in 2011.

Another limitation is that we did not categorize the patients into New York Heart Association (NYHA) functional classes. The functional class may have an essential impact on coping and QoL. In the present study, we do not know how many of the patients could be categorized into NYHA class III/IV. According to the regression analysis, the explained percentage of the variance was rather low for all models. One possible explanation is that the small sample size of patients may have limited the statistical power, especially because there were very few women with CHF. Another possible explanation is that we used mean substitution for missing values for the JCS scales. Yet the mean imputation may have underestimated the variance. However, to eliminate high correlations among the variables, multicollinearity tests (<2) were conducted among the independent variables. In the present study, no relationships between dimensions on the SF‐36 and the patient groups' characteristics were investigated, because the personal characteristics of the 1994 sample were not available. One strength of the study was the use of validated questionnaires (JCS‐60 and SF‐36) that had been used in previous Swedish studies (Lindqvist et al., 1998; Kristofferzon et al., 2011; Kristofferzon et al., 2005) obtaining nearly the same Cronbach′s alpha values. However, given that the Cronbach′s alpha values for some subscales of the JCS‐60 have previously been reported to be low (Kristofferzon, Lofmark, & Carlsson, 2005; Kristofferzon et al., 2011; Lindqvist et al., 1998), this could indicate a limitation in the reliability of the measurement and should be taken into consideration when interpreting the results.

## 5 Conclusion

In this study women used more problem‐focused as well as emotion‐focused coping strategies than men did. In addition, women in the patient group used coping the most. Compared with the Swedish general population, patients with CHF rated lower QoL. To deal with the psychological consequences of daily life, men with CHF and persons in the general Swedish population reported using both problem‐ and emotion‐focused coping. This indicates the need to investigate what meaning‐based coping can add to this research area and to clinical practice. However, nurses need to assess the coping strategies the patients use and encouraged patients abilities to improve their self‐ management.

## Conflict of interests

No conflict of interest has been declared by the authors.

## Authors' contributions

All authors have made contributions to the study design and to gathering and interpreting the data. AN and MLK participated in data collection, conducted the majority of the analyses and collaborated in writing the manuscript. All authors have been involved in critically revising the manuscript to ensure important intellectual content and have read and approved the final manuscript.

All the Authors have agreed on the final version and meet at least one of the following criteria [recommended by the ICMJE (http://www.icmje.org/recommendations/)]:

- substantial contribution to conception and design, acquisition of data or analysis and interpretation of data;
- drafting the article or revising it critically for important intellectual content.
